# A score for predicting colchicine resistance at the time of diagnosis in familial Mediterranean fever: data from the TURPAID registry

**DOI:** 10.1093/rheumatology/kead242

**Published:** 2023-05-25

**Authors:** Ezgi Deniz Batu, Seher Şener, Elif Arslanoglu Aydin, Emil Aliyev, İlknur Bagrul, Şeyma Türkmen, Özlem Akgün, Zeynep Balık, Ayşe Tanatar, Yağmur Bayındır, Zehra Kızıldağ, Rüya Torun, Aybüke Günalp, Taner Coşkuner, Rana İşgüder, Tuncay Aydın, Fatih Haşlak, Müşerref Kasap Cüceoğlu, Esra Esen, Ulaş Akçay, Özge Başaran, Aysenur Pac Kısaarslan, Fuat Akal, Deniz Yüce, Semanur Özdel, Mehmet Bülbül, Yelda Bilginer, Nuray Aktay Ayaz, Betül Sözeri, Özgür Kasapçopur, Erbil Ünsal, Seza Özen

**Affiliations:** Department of Pediatrics, Division of Rheumatology, Hacettepe University Faculty of Medicine, Ankara, Turkey; Department of Pediatrics, Division of Rheumatology, Hacettepe University Faculty of Medicine, Ankara, Turkey; Department of Pediatrics, Division of Rheumatology, Dr. Sami Ulus Maternity and Child Health and Diseases Research and Training Hospital, Ankara, Turkey; Department of Pediatrics, Division of Rheumatology, Hacettepe University Faculty of Medicine, Ankara, Turkey; Department of Pediatrics, Division of Rheumatology, Dr. Sami Ulus Maternity and Child Health and Diseases Research and Training Hospital, Ankara, Turkey; Department of Pediatrics, Division of Rheumatology, Umraniye Research and Training Hospital, Istanbul, Turkey; Department of Pediatrics, Division of Rheumatology, Istanbul University Faculty of Medicine, Istanbul, Turkey; Department of Pediatrics, Division of Rheumatology, Hacettepe University Faculty of Medicine, Ankara, Turkey; Department of Pediatrics, Division of Rheumatology, Istanbul University Faculty of Medicine, Istanbul, Turkey; Department of Pediatrics, Division of Rheumatology, Hacettepe University Faculty of Medicine, Ankara, Turkey; Department of Pediatrics, Division of Rheumatology, Dokuz Eylül University Faculty of Medicine, Izmir, Turkey; Department of Pediatrics, Division of Rheumatology, Dokuz Eylül University Faculty of Medicine, Izmir, Turkey; Department of Pediatrics, Division of Rheumatology, Istanbul University Cerrahpasa Faculty of Medicine, Istanbul, Turkey; Department of Pediatrics, Division of Rheumatology, Umraniye Research and Training Hospital, Istanbul, Turkey; Department of Pediatrics, Division of Rheumatology, Dokuz Eylül University Faculty of Medicine, Izmir, Turkey; Department of Pediatrics, Division of Rheumatology, Dokuz Eylül University Faculty of Medicine, Izmir, Turkey; Department of Pediatrics, Division of Rheumatology, Istanbul University Cerrahpasa Faculty of Medicine, Istanbul, Turkey; Department of Pediatrics, Division of Rheumatology, Hacettepe University Faculty of Medicine, Ankara, Turkey; Department of Pediatrics, Division of Rheumatology, Erciyes University Faculty of Medicine, Kayseri, Turkey; Department of Pediatrics, Division of Rheumatology, Umraniye Research and Training Hospital, Istanbul, Turkey; Department of Pediatrics, Division of Rheumatology, Hacettepe University Faculty of Medicine, Ankara, Turkey; Department of Pediatrics, Division of Rheumatology, Erciyes University Faculty of Medicine, Kayseri, Turkey; Department of Computer Engineering, Hacettepe University, Ankara, Turkey; Department of Preventive Oncology, Hacettepe University, Ankara, Turkey; Department of Pediatrics, Division of Rheumatology, Dr. Sami Ulus Maternity and Child Health and Diseases Research and Training Hospital, Ankara, Turkey; Department of Pediatrics, Division of Rheumatology, Dr. Sami Ulus Maternity and Child Health and Diseases Research and Training Hospital, Ankara, Turkey; Department of Pediatrics, Division of Rheumatology, Hacettepe University Faculty of Medicine, Ankara, Turkey; Department of Pediatrics, Division of Rheumatology, Istanbul University Faculty of Medicine, Istanbul, Turkey; Department of Pediatrics, Division of Rheumatology, Umraniye Research and Training Hospital, Istanbul, Turkey; Department of Pediatrics, Division of Rheumatology, Istanbul University Cerrahpasa Faculty of Medicine, Istanbul, Turkey; Department of Pediatrics, Division of Rheumatology, Dokuz Eylül University Faculty of Medicine, Izmir, Turkey; Department of Pediatrics, Division of Rheumatology, Hacettepe University Faculty of Medicine, Ankara, Turkey

**Keywords:** FMF, colchicine resistance, predictive score, paediatric FMF

## Abstract

**Objectives:**

Colchicine forms the mainstay of treatment in FMF. Approximately 5–10% of FMF patients are colchicine resistant and require anti-IL-1 drugs. We aimed to compare the characteristics of colchicine-resistant and colchicine-responsive patients and to develop a score for predicting colchicine resistance at the time of FMF diagnosis.

**Methods:**

FMF patients (0–18 years) enrolled in the Turkish Paediatric Autoinflammatory Diseases (TURPAID) registry were included. The predictive score for colchicine resistance was developed by using univariate/multivariate regression and receiver operating characteristics analyses.

**Results:**

A total of 3445 FMF patients [256 (7.4%) colchicine-resistant and 3189 colchicine-responsive) were included (female:male ratio 1.02; median age at diagnosis 67.4 months). Colchicine-resistant patients had longer, more frequent attacks and were younger at symptom onset and diagnosis (*P* < 0.05). Fever, erysipelas-like erythema, arthralgia, arthritis, myalgia, abdominal pain, diarrhoea, chest pain, comorbidities, parental consanguinity and homozygosity/compound heterozygosity for exon 10 *MEFV* mutations were significantly more prevalent among colchicine-resistant than colchicine-responsive patients (*P* < 0.05). Multivariate logistic regression analysis in the training cohort (*n* = 2684) showed that age at symptom onset, attack frequency, arthritis, chest pain and having two exon 10 mutations were the strongest predictors of colchicine resistance. The score including these items had a sensitivity of 81.3% and a specificity of 49.1%. In the validation cohort (*n* = 671), its sensitivity was 93.5% and specificity was 53.8%.

**Conclusion:**

We developed a clinician-friendly and practical predictive score that could help us identify FMF patients with a greater risk of colchicine resistance and tailor disease management individually at the time of diagnosis.

Rheumatology key messages

- In our large cohort of paediatric FMF patients (*N* = 3445), 256 (7.4%) children were colchicine resistant.
- Independent predictors of colchicine resistance were age at symptom onset, attack frequency, arthritis, chest pain and exon 10 *MEFV* mutations.
- The novel predictive score we proposed can assist in personalized management for FMF patients.

## Introduction

FMF is the most common monogenic recurrent fever syndrome [1]. The disease is characterized by febrile attacks of polyserositis accompanied by elevated acute phase inflammatory markers. Variants in the *MEFV* gene, which encodes for pyrin, are associated with FMF.

Colchicine has been the standard treatment for FMF over the last 50 years [1, 2]. It leads to the release of guanine nucleotide exchange factor H1 (GEF-H1), a RhoA activator, by binding to microtubules [3]. RhoA inhibits the overactivated mutant pyrin in FMF [3]. Although most FMF patients respond well to colchicine, colchicine resistance or intolerance constitutes a problem in 5–10% of patients [4]. The reason for colchicine resistance remains unknown. Anti-IL-1 drugs are currently used in the management of colchicine-resistant/intolerant FMF patients [5]. Recently an international consensus group defined colchicine resistance [6].

Several studies have analysed the differences between colchicine-resistant and colchicine-responsive FMF patients [7–10]. Features such as early symptom onset, greater attack frequency, longer attack duration, erysipelas-like erythema (ELE), myalgia/protracted febrile myalgia, arthritis, higher colchicine dose, high acute phase reactants and homozygosity for exon 10 *MEFV* mutations have been associated with colchicine-resistance so far [7–10]. In one of these studies, the authors attempted to develop a predictive score for colchicine resistance in FMF patients, however, the number of patients was limited (*n* = 104) [8].

Early prediction of colchicine resistance during FMF diagnosis would help physicians set up a personalized management and follow-up strategy. Also, we could provide appropriate prognostic information and present relevant therapeutic options to families. Furthermore, early intervention with biologic drugs during the follow-up could be beneficial in patients predicted to be colchicine resistant. In this study we aimed to compare the characteristics of colchicine-resistant and colchicine-responsive FMF patients and develop a score for predicting colchicine resistance based on the features of the patients at the time of diagnosis. This score does not serve as a definition of colchicine resistance to start biologic treatment, but serves as a tool to tailor patient management at the time of diagnosis.

## Patients and methods

The Turkish Paediatric Autoinflammatory Diseases (TURPAID) registry is the national web-based registry for paediatric autoinflammatory diseases in Turkey (https://turpaid.org). The registry was set up in 2021 and data entrance was initiated in February 2022. Paediatric rheumatologists from seven different centres in Turkey have entered data into the TURPAID database. Currently the registry includes data for 3510 paediatric patients with FMF. The baseline data includes demographic features, clinical manifestations, comorbidities, parental consanguinity, family history, genetic test results, results of radiologic and histopathological evaluations, treatment and outcome.

**Figure 1.**
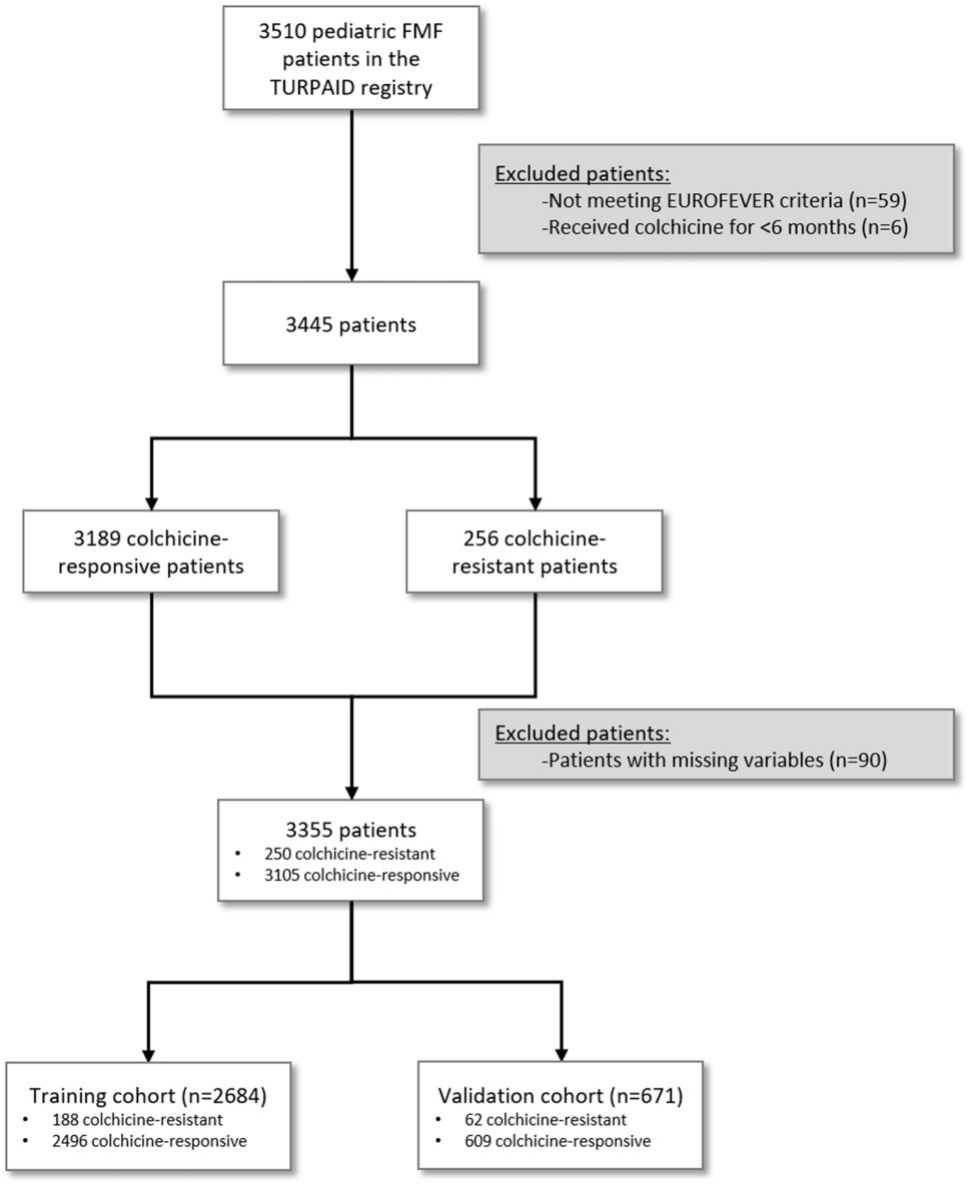
Flow diagram for paediatric patients with FMF in the TURPAID registry

The data for paediatric patients with FMF (0–18 years) were extracted from the TURPAID registry. Only FMF patients who met the EUROFEVER criteria for FMF [11] and received colchicine treatment for at least 6 months were included in this study (Fig. 1). It is noteworthy that the evaluated data of the patients belonged to the period before the initiation of colchicine treatment. Colchicine resistance was defined as having one or more attacks per month (over 3 months) and/or the presence of subclinical inflammation despite treatment with the maximum tolerable dose of colchicine [6]. The maximum colchicine dose was accepted as 2 mg/day in all participating centres.

The methods for genetic tests included *MEFV* single-gene analysis and gene panel analysis for several monogenic autoinflammatory diseases. The details of the genetic tests performed are presented in Supplementary Table S1, available at *Rheumatology* online.

The study was approved by the ethical committee of Hacettepe University (2020/17-38; approval date 20 October 2020) and was performed following the ethical standards laid down in the 1964 Declaration of Helsinki and its later amendments. Written informed consent was obtained from all parents/patients before inclusion in the study.

### Statistical analysis

Statistical analyses were performed using SPSS version 15 (IBM, Armonk, NY, USA). Descriptive analyses are presented using proportions, medians, means, s.d.s and interquartile ranges (IQRs) as appropriate. Categorical variables were compared by using the chi-squared test or Fisher’s exact test as appropriate. The numeric variables were investigated using visual (histogram, probability plots) and analytic methods (Kolmogorov–Smirnov) to determine whether they were normally distributed. The Student’s *t* and Mann–Whitney U tests were used to compare normally and non-normally distributed continuous variables.

Before regression analysis, patients with missing variables were excluded (Fig. 1) and the continuous variables were converted to binary variables using receiver operating characteristics (ROC) analysis. Then the cohort of patients (*n* = 3355) was divided into training and validation sets with a ratio of ≈80% to 20%, respectively, using the random sampling feature of the SPSS software (the ≈80%:20% ratio was valid both for colchicine-responsive and colchicine-resistant patients). Regression analysis was performed in the training set. To avoid the overpower bias due to the large sample size, only the variables with an unadjusted *P*-value <0.05 in the univariate analyses and the ones with clinical significance based on the authors’ discretion were utilized in multivariable logistic regression analysis. After establishing an initial model, we reduced the model by using backward elimination multivariate logistic regression analysis. A *P*-value <0.05 was considered statistically significant and the CI was 95%. The regression coefficient of each variable included in the final model was converted to a score by rounding to the nearest 0.5. The sum of these individual scores formed the predictive score for colchicine resistance. Then the best-performing cut-off value for predicting colchicine resistance was calculated using ROC analysis and the sensitivity and specificity were assessed afterwards. After this step, we tested the performance of the score in the validation cohort.

## Results

A total of 3445 FMF patients were included in this study from seven different centres in Turkey (Fig. 1). Colchicine resistance was observed in 256 (7.4%) patients (Table 1). The number of patients from each centre and the *MEFV* gene analysis results of the patients are presented in Supplementary Tables S2 and S3, respectively (available at *Rheumatology* online). Also, the comorbidities of the FMF patients are presented in Supplementary Table S4, available at *Rheumatology* online.

**Table 1.** The characteristics of colchicine-resistant and colchicine-responsive patients with FMF

Characteristics	All patients (*N* = 3445)	Colchicine resistant (*n* = 256)	Colchicine responsive (*n* = 3189)	*P*-value
Female, *n* (%)	1746 (50.7)	148 (57.8)	1598 (50.1)	0.018
Age at symptom onset, mean (s.d.), months	41.4 (53.4)	31.4 (42.6)	42.5 (55.2)	<0.001
Age at diagnosis, mean (s.d.), months	67.4 (66.6)	55.8 (48.4)	68.7 (67.1)	<0.001
Time from symptom onset to diagnosis, mean (s.d.), months	13.9 (25.4)	16.9 (24.5)	13.9 (25.6)	0.404
Attack duration, mean (s.d.), days	2.69 (1.03)	2.85 (1.1)	2.67 (1.02)	0.008
Attacks in 1 year before diagnosis, *n* (%)	10 (6)	12 (16)	10 (6)	<0.001
Comorbidity, *n* (%)	547 (15.9)	73 (28.5)	474 (14.9)	<0.001
Parental consanguinity, *n*/*N* (%)	559/3369 (16.6)	59/251 (23.5)	500/3118 (16.1)	0.009
Clinical findings, *n* (%)				
Fever	2901 (84.2)	234 (91.4)	2667 (83.6)	0.001
Malaise, *n*/*N* (%)	109/3430 (31.7)	14/255 (5.5)	95/3175 (29.9)	0.081
Erysipelas-like erythema	241 (6.9)	35 (13.7)	206 (6.5)	<0.001
Arthralgia	1590 (46.2)	154 (60.2)	1436 (45.1)	<0.001
Arthritis	676 (19.6)	90 (35.2)	586 (18.4)	<0.001
Myalgia	383 (11.1)	45 (17.6)	338 (10.6)	0.001
Abdominal pain	2962 (85.9)	232 (90.6)	2730 (85.6)	0.026
Nausea	127 (3.7)	12 (4.7)	115 (3.6)	0.541
Vomiting	187 (5.4)	19 (7.4)	168 (5.3)	0.251
Diarrhoea	213 (6.2)	26 (10.2)	187 (5.9)	0.027
Constipation	77 (2.2)	11 (4.3)	66 (2.1)	0.085
Chest pain	622 (18.1)	77 (30.1)	545 (17.1)	<0.001
Exon 10^a^	2158 (62.6)	234 (91.4)	1924 (60.3)	<0.001

a Homozygosity or compound heterozygosity for exon 10 *MEFV* mutations.

The median age at symptom onset and diagnosis were younger (31.4 *vs* 42.5 months and 55.8 *vs* 68.7 months, respectively), the mean duration of attacks was longer (2.85 *vs* 2.67 days) and the median number of attacks during 1 year before diagnosis were higher (12 *vs* 10) in the colchicine-resistant than colchicine-responsive patients (Table 1). Fever (91.4% *vs* 83.6%), ELE (13.7% *vs* 6.5%), arthralgia (60.2% *vs* 45.1%), arthritis (35.2% *vs* 18.4%), myalgia (17.6% *vs* 10.6%), abdominal pain (90.6% *vs* 85.6%), diarrhoea (10.2% *vs* 5.9%) and chest pain (30.1% *vs* 17.1%) were significantly more prevalent among colchicine-resistant patients compared with colchicine-responsive patients (*P* < 0.05). Also, comorbidity (28.5% *vs* 14.9%), parental consanguinity (23.5% *vs* 16.1%) and homozygosity or compound heterozygosity for exon 10 *MEFV* mutations (91.4% *vs* 60.3%) were more frequent in colchicine-resistant than responsive patients (*P* < 0.05) (Table 1).

**Figure 2.**
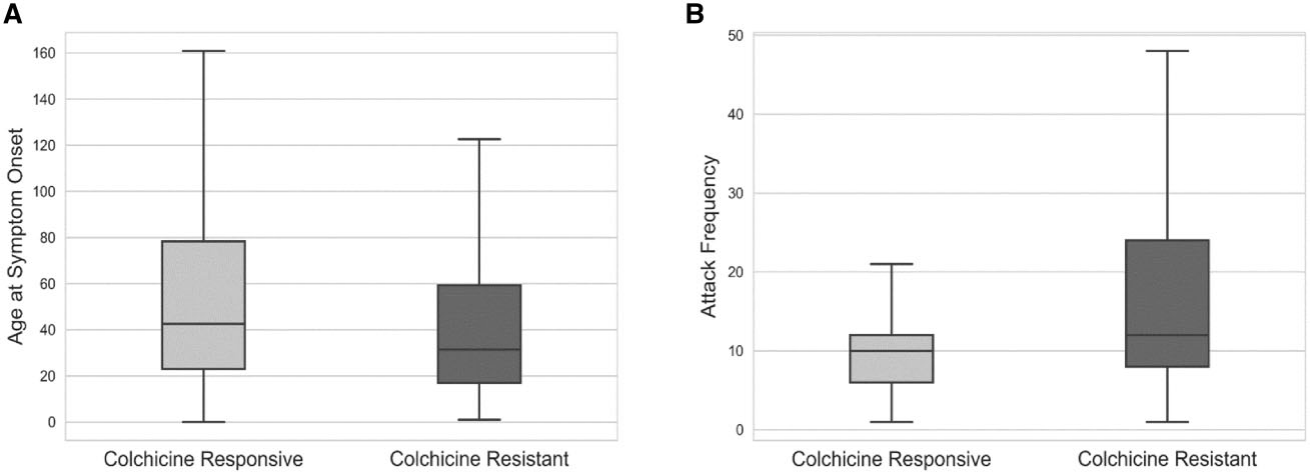
(**A**) Age at symptom onset (months) was younger (31.4 *vs* 42.5; *P* < 0.001) and (**B**) attack frequency (number of attacks during the year before the diagnosis) was higher (12 *vs* 10; *P* < 0.001) in colchicine-resistant FMF patients than colchicine-responsive ones

**Figure 3.**
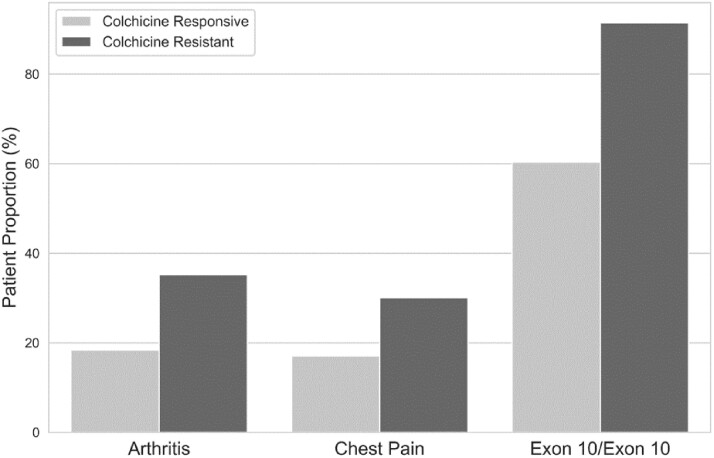
Arthritis (35.2% *vs* 18.4%), chest pain (30.1% *vs* 17.1%) and homozygosity or compound heterozygosity for exon 10 *MEFV* mutations (91.4% *vs* 60.3%) were more frequent among colchicine-resistant than colchicine-responsive FMF patients (*P* < 0.001 for all)

We divided the whole cohort (*N*= 3355 after excluding patients with missing variables) into training (*n* = 2684; 188 were colchicine resistant) and validation (*n* = 671; 62 were colchicine resistant) cohorts. We then identified the demographic and clinical characteristics that might have independent effects on colchicine resistance based on evaluations of statistical and clinical significance in univariate analyses in the training cohort. The initial logistic regression model built to detect the independent determinants of colchicine resistance using these variables is presented in Table 2. The final model identified that the independent determinants of colchicine resistance were the age at symptom onset ≤3 years [odds ratio (OR) 1.6 (95% CI 1.2, 2.2), *P* = 0.003], attack frequency before diagnosis ≥1/month [OR 1.8 (95% CI 1.3, 2.4), *P* < 0.001], arthritis [OR 2.3 (95% CI 1.6, 3.1), *P* < 0.001], chest pain [OR 1.6 (95% CI 1.1, 2.2), *P* = 0.009] and homozygosity or compound heterozygosity for exon 10 *MEFV* mutations [OR 4.4 (95% CI 2.8, 7.2), *P* < 0.001]. The differences between colchicine-responsive and colchicine-resistant patients in the whole cohort regarding these variables are presented in Figs 2 and 3.

**Table 2.** The evaluation of predictive factors for colchicine resistance in children with FMF using backward elimination multivariate logistic regression analysis

Variable	OR	95% CI	*P*-value
Initial model			
Female	1.2	0.8, 1.6	0.37
Age at symptom onset ≤3 years	1.6	1.2, 2.2	0.003
Parental consanguinity	1.4	0.9, 2	0.08
Erysipelas-like erythema	1.4	0.8, 2.2	0.21
Fever	1.5	0.9, 2.7	0.13
Attack frequency before diagnosis ≥1/month	1.7	1.2, 2.4	0.001
Arthritis	2.1	1.5, 2.9	<0.001
Myalgia	1.3	0.8, 2	0.26
Abdominal pain	1.3	0.8, 2.1	0.39
Diarrhoea	1.3	0.8, 2.3	0.30
Chest pain	1.5	1.1, 2.2	0.014
Exon 10^a^	4.3	2.7, 7	<0.001
Final model			
Age at symptom onset ≤3 years	1.6	1.2, 2.2	0.003
Attack frequency before diagnosis ≥1/month	1.8	1.3, 2.4	<0.001
Arthritis	2.3	1.6, 3.1	<0.001
Chest pain	1.6	1.1, 2.2	0.009
Exon 10^a^	4.4	2.8, 7.2	<0.001

a Homozygosity or compound heterozygosity for exon 10 *MEFV* mutations.

Based on the final regression model, we formulized a score to predict the colchicine resistance and the logistic regression coefficients and proposed subscores for each characteristic are presented in Table 3. The maximum score was 4. The best-performing cut-off value of the sum that discriminated colchicine-resistant patients from the colchicine-responsive patients was ≥2. The area under the curve (AUC) was 0.731 [s.e. 0.018 (95% CI 0.695, 0.766) , *P* < 0.001] in the ROC analysis and the sensitivity and specificity of the score were 81.3% and 49.1%, respectively. When we tested the performance of this score in the validation cohort (*n* = 671), its sensitivity was 93.5% and specificity was 53.8%. The user-friendly web application for the proposed score is available online (https://turpaid.streamlit.app/).

**Table 3.** Score for predicting colchicine-resistant disease course in patients with FMF^a^

Feature	β	s.e.	Points per item
Age at symptom onset ≤3 years	0.47	0.16	0.5
Attack frequency before diagnosis ≥1 attack/month	0.57	0.16	0.5
Arthritis	0.82	0.17	1
Chest pain	0.46	0.18	0.5
Exon 10^b^	1.5	0.24	1.5

a The maximum score is 4. A threshold of ≥2 predicts colchicine resistance with a sensitivity of 81.3% and 93.5% and a specificity of 49.1% and 53.8% in the training and validation cohorts, respectively.

b Homozygosity or compound heterozygosity for exon 10 *MEFV* mutations.

## Discussion

In the presented study, which includes one of the largest paediatric FMF cohorts of 3445 patients, 7.4% of the patients were colchicine resistant. The age at symptom onset was younger and attacks were longer and more frequent and female gender, comorbidities, parental consanguinity, exon 10/exon 10 *MEFV* mutations, fever, ELE, myalgia, arthralgia, arthritis, abdominal pain, chest pain and diarrhoea were more common among colchicine-resistant patients as compared with those who were colchicine responsive. We divided this large cohort into training and validation cohorts and a score was developed in the training cohort including the independent predictors of colchicine resistance as the age of symptom onset ≤3 years, attack frequency before diagnosis ≥1 attack/month, arthritis, chest pain and homozygosity or compound heterozygosity for exon 10 *MEFV* mutations. This score has a sensitivity of 81.3% and 93.5% and a specificity of 49.1% and 53.8% in the training and validation cohorts, respectively, for predicting colchicine resistance at the time of diagnosis in FMF patients.

The rate of colchicine resistance (7.4%) of our cohort was comparable to the previously reported frequencies of 5–10% in the literature [12]. In a recent cohort study including 3554 FMF patients, ≈4.5% of patients were colchicine resistant [10].

To date, several features of FMF patients have been suggested to predict colchicine resistance. M694V homozygosity has been among the most popular [10, 13, 14]. M694V homozygosity was observed in >70% of colchicine-resistant FMF patients in the literature [5, 15–17]. In the score we proposed, having two exon 10 *MEFV* mutations has the highest item value, and >90% of colchicine-resistant patients were homozygous or compound heterozygous for exon 10 *MEFV* mutations in our cohort. Along the same lines, Ozturk *et al.* [10] demonstrated that M694V homozygosity was associated with colchicine resistance. In the EULAR guideline for genetic diagnosis of FMF, the authors mentioned that exon 10 *MEFV* mutations, especially the ones at positions 680–694 (such as M680I or M694V), were associated with a greater risk of severe disease [14].

Early onset of symptoms and more frequent attacks are commonly indicated as the features of severe disease in FMF [18–21]. We previously demonstrated that younger age at symptom onset was associated with more severe disease and M694V homozygosity [18]. More frequent attacks were among the independent predictors of colchicine-resistant FMF in two previous studies, as well [8, 22].

Chest pain is observed in FMF patients as a part of serosal inflammation. It is usually due to pleuritis and unilateral, but pericarditis could also cause retrosternal chest pain [1]. Chest pain was present in 18% of patients in our cohort. Similarly, 20% of FMF patients had chest pain during attacks in the large cohort of Ozturk *et al.* [10]. Chest pain is frequently associated with severe disease, persistent inflammation and colchicine resistance in FMF in the literature [10, 13, 23]. The reason is probably the more profound serosal inflammation in severe FMF. The frequency of chest pain is 35–60% in colchicine-resistant FMF patients in the literature, which may differ according to the ethnic group [7, 8, 15, 17, 24]; this frequency was 30% in our study. Another factor could be that chest pain is more frequent among patients with exon 10 mutations [25, 26], and the majority of colchicine-resistant patients have exon 10 *MEFV* mutations.

Joint inflammation is also a common feature of FMF. Acute arthritis could occur during attacks, but also FMF patients more frequently have chronic arthritis [1]. Arthritis was observed in ≈30–50% of FMF patients who were colchicine resistant [7, 17, 24] and was present in 35% of colchicine-resistant patients in our cohort. In a recent study by Omma *et al.* [24], the only significant difference between colchicine-resistant and colchicine-responsive FMF patients was the higher frequency of arthritis among colchicine-resistant patients. Gezgin-Yıldırım *et al.* [13] recently examined the factors predicting persistent inflammation in FMF. The multivariate analysis established arthritis along with M694V homozygosity, chest pain, leg pain, colchicine resistance, ELE, inflammatory comorbidities, high Pras score, early disease onset and long attack duration as independent predictors of persistent inflammation.

There are a few studies that have analysed the predictive factors for colchicine resistance in FMF; however, none of these focused on features present at the time of FMF diagnosis. Sahin *et al.* [22] have shown attack frequency and high erythrocyte sedimentation rate as independent predictors of colchicine resistance in paediatric FMF patients (*n* = 88). In another study by Erdem Gursoy *et al.* [7], longer attack duration and arthritis were the only independent predicting factors (*n* = 118). Neutropenia, duration of fever after colchicine treatment, attack frequency before colchicine treatment, skin rash/ELE, the dose of colchicine and C-reactive protein levels were included in the model score proposed by Mosad Mosa *et al.* [8] (*n* = 104). However, the sample size was <150 patients in all previous studies analysing the predictive factors for colchicine resistance in FMF. The major strength of our study is the large sample size (*N* = 3445). Moreover, we focused on the features present at the time of FMF diagnosis. This is important since it gives the possibility of predicting colchicine resistance at the beginning of colchicine treatment. Also, the score we proposed is easy to use and practical. Physicians could use the online application of the score to skip tiring mathematical calculations during their daily clinical routine, thus its clinical implementation should be easy.

There are several limitations in this study. The large sample size might have introduced a risk for overpower bias. In order to avoid this bias, we utilized a strict criterion (*P* < 0.05 instead of the classically used *P* < 0.15–0.20) while selecting statistically significant features in the univariate analysis. Moreover, we prioritized the clinical significance evaluated by the authors experienced in FMF. Another point is that colchicine compliance was not evaluated. Thus non-compliance could be an issue in patients who did not respond to colchicine treatment. Also, colchicine-responsive patients might have been lost to follow-up more frequently than colchicine-resistant ones, which may cause a slight bias in selecting colchicine-resistant patients when entering data into the registry. Lastly, we were unable to compare the maximum ‘tolerable’ dose of colchicine between colchicine-responsive and colchicine-resistant patients since these data were not recorded in the database.

In conclusion, we developed a physician-friendly practical score for predicting colchicine resistance at the time of diagnosis in FMF. This score could complement early intervention in colchicine-resistant FMF patients and the implementation of personalized treatment strategies in the management of FMF patients.

## Supplementary Material

kead242_Supplementary_Data

## Data Availability

The dataset used for this study is available from the corresponding author upon reasonable request.
