# Supplementary material for: A score for predicting colchicine resistance at the time of diagnosis in familial Mediterranean fever: data from the TURPAID registry
Source: Rheumatology (Oxford). 2023 May 25;63(3):791–7. doi: 10.1093/rheumatology/kead242 (PMC10907807; doi:10.1093/rheumatology/kead242)
Supplement: kead242_Supplementary_Data [file kead242_supplementary_data.docx]

**Supplementary Table S1.** The methods for genetic tests performed in patients with familial Mediterranean fever (FMF)

| **Single gene (MEFV) analysis, n (%)**  Sanger sequencing of exons  Screening of point mutations with Sanger sequencing  NGS  PCR | 2933 (85.1)  1437 (49)  905 (30.8)  393 (13.4)  198 (6.8) |
| --- | --- |
| **Analysis of a gene panel associated with monogenic SAIDs, n (%)**  NGS  Sanger sequencing of exons  Screening of point mutations with Sanger sequencing | 512 (14.9)  501 (97.9)  7 (1.4)  4 (0.7) |

*NGS, next generation sequencing; PCR,* *polymerase chain reaction; SAID, systemic autoinflammatory diseases*

**Supplementary Table S2.** Number of familial Mediterranean fever (FMF) patients from different pediatric rheumatology centers in Turkey (total n=3445)

| **Name of the center** | **FMF patients, n (%)** |
| --- | --- |
| Hacettepe University Faculty of Medicine | 1181 (34.3) |
| Dr. Sami Ulus Maternity and Child Health and Diseases Research and Training Hospital | 618 (17.9) |
| Dokuz Eylül University Faculty of Medicine | 500 (14.5) |
| Umraniye Research and Training Hospital | 402 (11.6) |
| Istanbul University Faculty of Medicine | 354 (10.3) |
| Istanbul University Cerrahpasa Faculty of Medicine | 209 (6.1) |
| Erciyes University Faculty of Medicine | 181 (5.3) |

**Supplementary Table S3.** *MEFV* genotypes of patients with familial Mediterranean fever (FMF) (total n=3445)

| ***MEFV* genotype** | **n (%)** |
| --- | --- |
| M694V/M694V | 1040 (30.2) |
| M694V/- | 597 (17.3) |
| M694V/M680I | 337 (9.8) |
| M694V/V726A | 262 (7.6) |
| M694V/E148Q | 142 (4.1) |
| M694V/R202Q | 122 (3.5) |
| V726A/- | 118 (3.4) |
| M680I/V726A | 107 (3.2) |
| M680I/- | 104 (3.1) |
| M680I/M680I | 103 (2.9) |
| M694V/R761H | 54 (1.6) |
| M694V/M694V/R202Q/R202Q | 54 (1.6) |
| M694V/V726A | 30 (0.87) |
| E148Q/E148Q | 27 (0.78) |
| V726A/V726A | 25 (0.72) |
| M680I/E148Q | 22 (0.63) |
| V726A/E148Q | 21 (0.61) |
| E148Q/P369S | 18 (0.52) |
| R761H/- | 17 (0.49) |
| M694V/M694I | 16 (0.46) |
| M680I/R761H | 15 (0.43) |
| M694V/R202Q/R202Q | 15 (0.43) |
| M694V/M680I/R202Q | 14 (0.41) |
| M694V/M694V/R202Q | 12 (0.35) |
| V726A/R202Q | 11 (0.32) |
| K695R/- | 9 (0.26) |
| M680I/R202Q | 7 (0.21) |
| M694V/V726A/R202Q | 7 (0.21) |
| P369S/R408Q | 7 (0.21) |
| R761H/R761H | 7 (0.21) |
| V726A/R761H | 7 (0.21) |
| M694V/E148Q/R202Q | 6 (0.17) |
| M694V/R761H | 6 (0.17) |
| M694V/A744S | 6 (0.17) |
| V726A/A744S | 5 (0.14) |
| E167D/F479L | 4 (0.12) |
| K695R/R202Q | 4 (0.12) |
| M694V/V726A/R202Q | 4 (0.12) |
| R761H/E148Q | 4 (0.12) |
| K695R/E148Q | 3 (0.09) |
| M680I/K695R | 3 (0.09) |
| M694V/P369S | 3 (0.09) |
| V726A/F479L | 3 (0.09) |
| V726A/K695R | 3 (0.09) |
| A744S/E148Q | 2 (0.05) |
| F479L/- | 2 (0.05) |
| M680I/A744S | 2 (0.05) |
| M694I/M680I | 2 (0.05) |
| M694V/E148Q/P369S | 2 (0.05) |
| M694V/E167D | 2 (0.05) |
| M694V/K695R | 2 (0.05) |
| M694V/M680I/E148Q | 2 (0.05) |
| M694V/M680I/R202Q/R202Q | 2 (0.05) |
| M694V/M694DEL | 2 (0.05) |
| M694V/M694V/E148Q/E148Q | 2 (0.05) |
| M694V/R408Q | 2 (0.05) |
| V726A/P369S | 2 (0.05) |
| E148Q/P369S/E230K | 1 (0.03) |
| E148Q/P369S/R408Q | 1 (0.03) |
| E148Q/R408Q | 1 (0.03) |
| E167D/E167D/F479L/F479L | 1 (0.03) |
| M680I/E167D | 1 (0.03) |
| M680I/P369S | 1 (0.03) |
| M680I/R143P | 1 (0.03) |
| M680I/V726A/R202Q | 1 (0.03) |
| M694DEL/E148Q | 1 (0.03) |
| M694DEL/M680I | 1 (0.03) |
| M694DEL/M694DEL | 1 (0.03) |
| M694I/E148Q | 1 (0.03) |
| M694I/R761H | 1 (0.03) |
| M694V/A744S/R202Q | 1 (0.03) |
| M694V/E148Q/E148Q | 1 (0.03) |
| M694V/E167D/E167D/F479L | 1 (0.03) |
| M694V/I591T | 1 (0.03) |
| M694V/M680I/V726A | 1 (0.03) |
| M694V/M694V/E148Q | 1 (0.03) |
| M694V/P369S/P369S | 1 (0.03) |
| M694V/P369S/R202Q | 1 (0.03) |
| M694V/P369S/R408Q | 1 (0.03) |
| M694V/R761H/R202Q | 1 (0.03) |
| M694V/V726A/E148Q | 1 (0.03) |
| M69V/R761C | 1 (0.03) |
| P369S/A744S | 1 (0.03) |
| R501C/P369S/R408Q/R314C | 1 (0.03) |
| R761H/P369S | 1 (0.03) |
| R761H/R202Q | 1 (0.03) |
| V726A/E148Q/R202Q | 1 (0.03) |
| V726A/E167D | 1 (0.03) |
| V726A/E251K | 1 (0.03) |
| V726A/F479L/E167D | 1 (0.03) |
| V726A/P369S/R202Q | 1 (0.03) |
| V726A/R408Q | 1 (0.03) |
| V726A/T267I | 1 (0.03) |
| V726A/V469L | 1 (0.03) |
| V726A/V704I | 1 (0.03) |

**Supplementary Table S4.** Comorbidities in colchicine-resistant and colchicine-responsive patients with familial Mediterranean fever (FMF)

| **n (%)** | **Colchicine-resistant patients (n=73)** | **Colchicine-responsive**  **patients (n=474)** |
| --- | --- | --- |
| JIA | 19 (26) | 120 (25.3) |
| IgA vasculitis | 9 (12.3) | 65 (13.7) |
| Hepatosteatosis | 4 (5) | 6 (1.2) |
| Renal failure | 4 (5) | 0 |
| IBD | 3 (4.1) | 20 (4.2) |
| PFAPA syndrome | 2 (2.7) | 28 (5.9) |
| Asthma | 2 (2.5) | 27 (5.6) |
| Obesity | 2 (2.5) | 14 (2.9) |
| Epilepsy | 2 (2.5) | 8 (1.6) |
| VUR | 2 (2.5) | 6 (1.2) |
| Congenital GIS pathologies | 2 (2.5) | 5 (1) |
| ARF | 2 (2.5) | 3 (0.6) |
| Hearing loss | 2 (2.5) | 2 (0.4) |
| Proteinuria | 2 (2.5) | 1 (0.2) |
| Factor 7 deficiency | 2 (2.5) | 0 |
| Hypothyroidism | 1 (1.3) | 11 (2.3) |
| GH deficiency | 1 (1.3) | 9 (1.8) |
| Selective IgA deficiency | 1 (1.3) | 8 (1.6) |
| PCOS | 1 (1.3) | 6 (1.2) |
| Uveitis | 1 (1.3) | 6 (1.2) |
| PAN | 1 (1.3) | 4 (0.8) |
| Thalassemia minor | 1 (1.3) | 9 (1.8) |
| Anxiety disorder | 1 (1.3) | 3 (0.6) |
| Celiac disease | 1 (1.3) | 3 (0.6) |
| CNO | 1 (1.3) | 3 (0.6) |
| Cerebral palsy | 1 (1.3) | 2 (0.4) |
| Chronic diarrhea | 1 (1.3) | 2 (0.4) |
| Hypertension | 1 (1.3) | 2 (0.4) |
| Nutcracker syndrome | 1 (1.3) | 2 (0.4) |
| Enuresis nocturna | 1 (1.3) | 1 (0.2) |
| Restrictive cardiomyopathy | 1 (1.3) | 1 (0.2) |
| Turner syndrome | 1 (1.3) | 1 (0.2) |
| Hypoparathyroidism | 1 (1.3) | 0 |
| Migraine | 1 (1.3) | 0 |
| Nephrotic syndrome | 1 (1.3) | 0 |
| Recurrent urinary tract infection | 0 | 12 (2.5) |
| Attention deficit and hyperactivity disorder | 0 | 10 (2.1) |
| Diabetes mellitus | 0 | 8 (1.6) |
| Short stature | 0 | 6 (1.2) |
| Other immunodeficiencies | 0 | 5 (1) |
| Nephrolithiasis | 0 | 5 (1) |
| Malignancy | 0 | 5 (1) |
| Hydronephrosis | 0 | 4 (0.8) |
| Precox puberty | 0 | 4 (0.8) |
| Atopic dermatitis | 0 | 3 (0.6) |
| Autism | 0 | 3 (0.6) |
| Behcet's disease | 0 | 3 (0.6) |
| Food allergy | 0 | 3 (0.6) |
| Growth restriction | 0 | 3 (0.6) |
| Psoriasis | 0 | 3 (0.6) |
| Adrenal insufficiency | 0 | 2 (0.4) |
| Arrhythmia | 0 | 2 (0.4) |
| ASD | 0 | 2 (0.4) |
| Autoimmune hepatitis | 0 | 2 (0.4) |
| Bronchiectasis | 0 | 2 (0.4) |
| G6PD deficiency | 0 | 2 (0.4) |
| Gastritis | 0 | 2 (0.4) |
| Hemangioma | 0 | 2 (0.4) |
| Mental retardation | 0 | 2 (0.4) |
| Mitral regurgitation | 0 | 2 (0.4) |
| VSD | 0 | 2 (0.4) |
| ITP | 0 | 2 (0.4) |
| Recurrent anal abscess | 0 | 1 (0.2) |
| Anemia | 0 | 1 (0.2) |
| Aortic regurgitation | 0 | 1 (0.2) |
| Aortic stenosis | 0 | 1 (0.2) |
| Asplenia | 0 | 1 (0.2) |
| AV fistula | 0 | 1 (0.2) |
| AVSD | 0 | 1 (0.2) |
| Cataract | 0 | 1 (0.2) |
| Cholelithiasis | 0 | 1 (0.2) |
| Cholestatic hepatitis | 0 | 1 (0.2) |
| Chronic liver disease | 0 | 1 (0.2) |
| Chylomicron retention disease | 0 | 1 (0.2) |
| Congenital heart disease | 0 | 1 (0.2) |
| Craniosynostosis | 0 | 1 (0.2) |
| Cutaneous lipoma | 0 | 1 (0.2) |
| Cyclic neutropenia | 0 | 1 (0.2) |
| Developmental dysplasia of the hip | 0 | 1 (0.2) |
| Down syndrome | 0 | 1 (0.2) |
| Fatty acid oxidation defect | 0 | 1 (0.2) |
| Fibromyalgia | 0 | 1 (0.2) |
| Fructose intolerance | 0 | 1 (0.2) |
| Gallbladder polyps | 0 | 1 (0.2) |
| Gastroesophageal reflux | 0 | 1 (0.2) |
| Gilbert syndrome | 0 | 1 (0.2) |
| Hemangioendothelioma | 0 | 1 (0.2) |
| Hydrocephalus | 0 | 1 (0.2) |
| Hyperlipidemia | 0 | 1 (0.2) |
| Hypofibrinogenemia | 0 | 1 (0.2) |
| Hypotonic infant | 0 | 1 (0.2) |
| IgA Nephropathy | 0 | 1 (0.2) |
| Kawasaki disease | 0 | 1 (0.2) |
| Keratosis pilaris | 0 | 1 (0.2) |
| Legg-Calve-Perthes disease | 0 | 1 (0.2) |
| MIS-C | 0 | 1 (0.2) |
| Microcephaly | 0 | 1 (0.2) |
| MVP | 0 | 1 (0.2) |
| Myasthenia gravis | 0 | 1 (0.2) |
| Non-Hodgkin lymphoma | 0 | 1 (0.2) |
| PDA | 0 | 1 (0.2) |
| Post-traumatic stress disorder | 0 | 1 (0.2) |
| Pubertas tarda | 0 | 1 (0.2) |
| Raynaud's phenomenon | 0 | 1 (0.2) |
| Scleroderma | 0 | 1 (0.2) |
| Sever's disease | 0 | 1 (0.2) |
| Situs inversus | 0 | 1 (0.2) |
| Urticaria | 0 | 1 (0.2) |
| Von Willebrand disease | 0 | 1 (0.2) |
| Wilson disease | 0 | 1 (0.2) |

*ARF, acute rheumatic fever; ASD, atrial septal defect; AV, atrioventricular; AVSD, atrioventricular septal defect; CNO, chronic nonbacterial osteomyelitis; GIS, gastrointestinal system; GH, growth hormone; G6PD, glucose-6-phosphate dehydrogenase; IgA, immunoglobulin A; ITP, idiopathic thrombocytopenic purpura; JIA, juvenile idiopathic arthritis; MIS-C; multisystem inflammatory syndrome in children; MVP, mitral valve prolapse; PAN, polyarteritis nodosa; PCOS, polycystic ovary syndrome; PFAPA, periodic fever, aphthous stomatitis, pharyngitis, adenitis; PDA, patent ductus arteriosus; VSD, ventricular septal defect; VUR, vesicoureteral reflux*
